# Vestibular event monitoring in acute posterior circulation stroke: from emergency room to stroke unit and beyond

**DOI:** 10.1007/s00415-025-13163-4

**Published:** 2025-07-15

**Authors:** Gülden Akdal, Benjamin Nham, Belinda Kwok, Pinar Özcelik, Andrew Bradshaw, Chao Wang, Gábor Michael Halmágyi, Miriam S Welgampola

**Affiliations:** 1 Neurology Department, Dokuz Eylül University Hospital, Izmir, Turkey; 2 Royal Prince Alfred Hospital Medical Centre, Sydney, Australia; 3 Neurology Department, Royal Prince Alfred Hospital, Sydney, Australia

Dear Sirs,

The HINTS clinical examination (Head Impulse, Nystagmus, Tests of Skew) surpasses early MRI as a discriminator of the two common causes of acute vestibular syndrome (AVS): vestibular neuritis and acute posterior circulation stroke (PCS) [1]. In the emergency room setting capturing and measuring nystagmus characterisctics with videonystagmography (VNG) improves accuracy of the HINTS examination [2]. Patients presenting with AVS due to acute PCS could be expected to have nystagmus. Some patients have central types of nystagmus such as gaze-evoked, direction-changing horizontal nystagmus or vertical/torsional nystagmus others have *peripheral-looking* unidirectional horizontal nystagmus and some have no nystagmus at all [2–6]. We wondered if some acute PCS patients who have no nystagmus at presentation will later develop nystagmus, or if a nystagmus that occurs at presentation later converts to a different type nystagmus. To do this we extended our VNG examination after patients were admitted from the emergency room to the neurology ward.

The VNG method we used here was the same as in our previous report [2]. With this method, we studied nystagmus in 80 acute PCS patients, presenting with vertigo or imbalance or both. Five patients had a peripheral “HINTS plus” examination at presentation with *peripheral-looking* unidirectional nystagmus beating away from the side of the positive head impulse test. Two of these 5 patients had PICA infarcts, one had a pontine infarct, one had a anterior inferior cerebellar territory infarct and one had a brainstem perforator infarct.

We studied each patient once a day, on at least 2 extra days after presentation, up to the 9th day after presentation. A total of 238 VNG recordings were made, 181 (76%) in the first 3 days. Stroke territories were: posterior inferior cerebellar artery (PICA) in 33 patients, anterior inferior cerebellar artery (AICA) in 5, superior cerebellar artery (SCA) in 2, brainstem perforators in 21, posterior cerebral artery in 2, middle cerebral artery in 2, or multiple arterial territories in 15. We assumed that patients showing only middle or posterior cerebral artery strokes also had brainstem or cerebellar strokes that escaped imaging,

Of the 80 patients, 47 had nystagmus at presentation which was, broadly speaking, one of 3 types: (1) Gaze-evoked, direction-changing, horizontal nystagmus (14 patients); on day 1 their nystagmus slow phase velocity (SPV) ranged from 1.4° to 16.2°/s. (2) Primary-position vertical nystagmus (5 upbeating and 5 down beating), some with a torsional component; their vertical SPV ranged from 1.2° to 43.9°/s; (3) Unidirectional horizontal *peripheral looking* nystagmus, most with a slight torsional component (23 patients); on day 1 their horizontal SPV ranged from 1.3° to 13.0°/s. (We did not measure torsional component of nystagmus.) The type of nystagmus did not change on subsequent recordings, remaining consistent with the initial peripheral looking or central-looking appearance. Of these 47 patients presenting with nystagmus, 3 had only vertigo at presentation, 7 had only imbalance and 37 had both.

Of the 80 patients, 33 had no nystagmus at presentation and 21 of them still had no nystagmus in subsequent recordings up to day 9, whereas 12 did develop nystagmus some as early as on day 2. Of these 12 patients with delayed nystagmus, 5 developed peripheral-looking nystagmus and 7 developed central-looking nystagmus—horizontal or vertical or both. Of these 33 patients presenting without nystagmus, 3 had only vertigo at presentation, 3 had only imbalance and 27 had both. There was no overall difference in the presence or absence of any other posterior fossa neurological signs such as sensory or motor impairment in cranial nerves or limbs, or in the neuro-vascular territories involved, between patients who developed central patterns of nystagmus, those who developed peripheral patterns of nystagmus and those who developed no nystagmus (see Fig. 1).

**Fig. 1:**
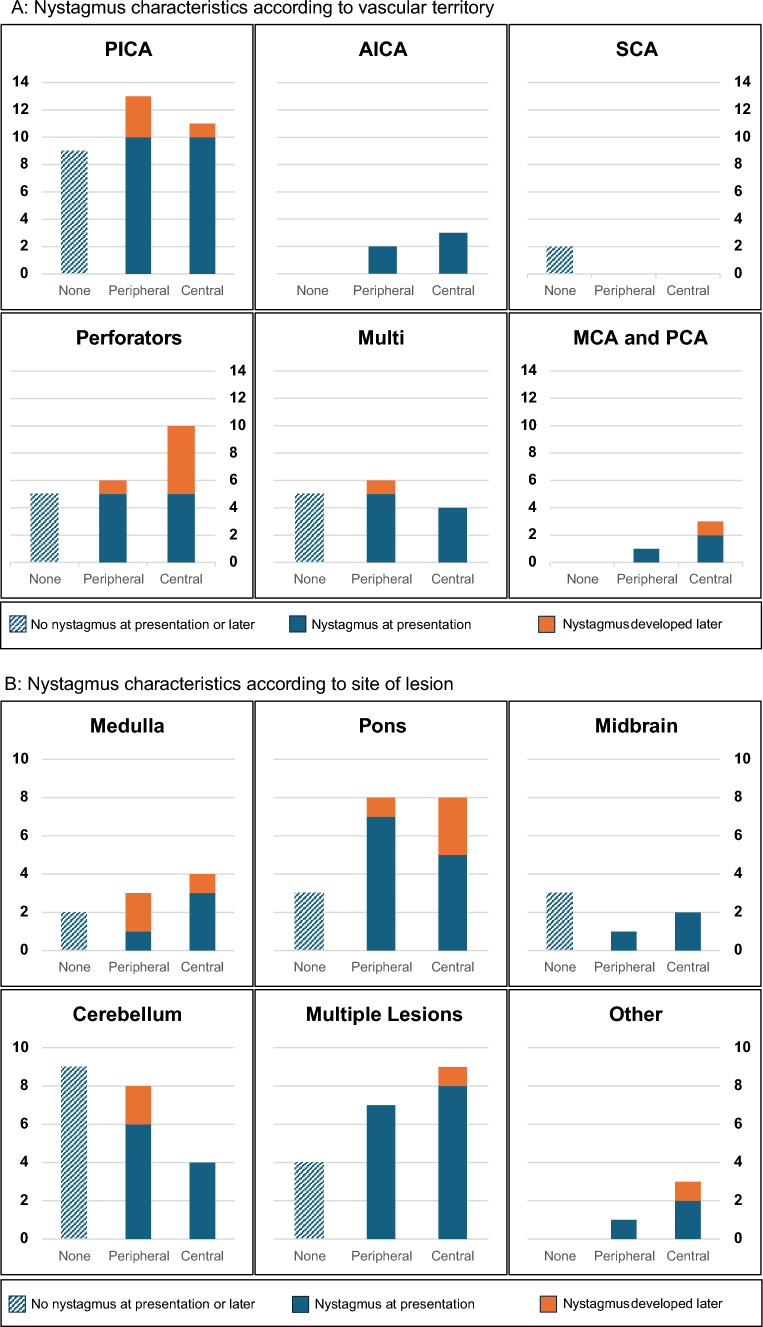
In both figures the solid blue bars indicate nystagmus at presentation; the orange caps on the solid blue bars indicate the delayed nystagmus that developed up to 9 days after presentation. The light blue dotted bars labelled NONE indicate patients who never developed nystagmus either on presentation or later. **A** Nystagmus characteristics according vascular territory involved.* PICA* Posterior inferior cerebellar artery, *AICA* anterior inferior cerebellar artery, *SCA* superior cerebellar artery, *Perforators* brainstem perforators, *Multi* multiple territories, *MCA* *and* *PCA* middle and posterior cerebral artery—presumably there were associated unimaged infarcts in the brainstem or cerebellum. Here, as in the text, “peripheral’ means peripheral-looking, unidirectional horizontal nystagmus. **B** Nystagmus characteristics according to site of lesion: medulla (all dorsolateral) pons, midbrain, cerebellum, multiple lesions and other

These observations confirm that in a patient presenting with AVS or acute imbalance, the absence of nystagmus or any other neurologic sign, does not mean that the patient has not had a PCS [2, 5, 6]. Absence of evidence is not evidence of absence. Here we show than even if no nystagmus is found at presentation, even with VNG, nystagmus could develop the next day or even later, even without obvious extension of the stroke. Unless there are convincing signs of an acute unilateral peripheral vestibular lesion such as unidirectional horizontal nystagmus with a positive head impulse test opposite to the quick phase direction of the nystagmus [2], we suggest that it is safer to admit, observe and re-examine such patients in the neurology ward, at least until a DWI MRI is done 2–3 days later.

## Data Availability

Data is available for perusal.
